# Exploring motivations of peer supporters for caregivers of patients with hematological malignancies—a qualitative study

**DOI:** 10.1007/s00520-025-09283-2

**Published:** 2025-02-26

**Authors:** Cæcilie Borregaard Myrhøj, Iben Husted Nielsen, Camilla Louise Visler, Kristina Holmegaard Nørskov, Karin Piil, Mary Jarden

**Affiliations:** 1 Department of Hematology, Copenhagen University Hospital, Copenhagen, Denmark; 2 Department of Hematology, Zealand University Hospital, Roskilde, Denmark; 3 Department of Clinical Medicine, University of Copenhagen, Copenhagen, Denmark; 4 Department of Oncology, Copenhagen University Hospital, Copenhagen, Denmark; 5 Department of People and Technology, Roskilde University, Roskilde, Denmark

**Keywords:** Peer support, Motivations, Hematological malignancies

## Abstract

**Purpose:**

Social support interventions, particularly peer support from former family caregivers, offer unique assistance to caregivers of newly diagnosed patients. Since voluntary peer support is driven by personal choice, understanding the motivations for participating and how motivation evolves over time is essential. This study explores the motivations for becoming a peer support provider for family caregivers of patients with hematological malignancies and how motivation changes over time.

**Methods:**

This qualitative study encompasses 18 semi-structured interviews with family caregivers providing peer support (*N* = 11) at two time points: (1) just after certification as peer supporters but prior to starting the peer support program and (2) at the 6-week mark of their 12-week peer support program. Interpretive phenomenological analysis was used to analyze the data.

**Results:**

Motivation for participating in a voluntary peer-to-peer support program as a peer support provider center on four main themes: “Driven by past experiences”, “Moral obligation is intrinsic to identity”, “Meaningful use of personal experiences”, and “Guiding new caregivers”. Motivation was influenced during the support program, particularly by the presence or absence of feedback from family caregivers and by participation in network meetings with other peer support providers.

**Conclusions:**

This study emphasizes the importance of networking meetings for family caregiver peer support providers, as they facilitate exchange of knowledge and insights and discussion of challenges and rewards and provide an ongoing support and motivation. It also highlights the untapped potential of volunteering to provide unique social support benefiting both the family caregiver recipient of support and the support provider.

## Introduction

Hematological malignancies are life-threatening diseases characterized by high mortality, increased risk of complications due to bone marrow failure, significant symptom burden, and intensive chemotherapy regimens [1–3]. Family caregivers play a pivotal role in supporting patients during treatment. However, being a caregiver may impose a substantial burden, unmet needs, and psychological distress [4] with high psychosocial morbidity [5]. Previous research shows that caregivers have a significant need for social support [6–9].

Evidence suggests that psychosocial interventions generate positive outcomes on mental health and well-being [10–12]. Psychosocial support, such as peer-to-peer support, is defined as support provided by a person with similar lived experiences [13]. Thus, peer-to-peer support offers unique assistance to patients and caregivers through authentic experiences and personal insights, delivering informational, emotional, and practical help that supplements support from healthcare professionals and social networks [14–18].

However, few studies have examined peer support interventions in caregivers of patients with cancer, particularly within hematological malignancies [19, 20]. Peer support is often based on volunteer work from former patients or caregivers who “work” for organizations without monetary compensation [21]. Understanding what motivates individuals to participate in volunteer social support activities is essential for ensuring the effectiveness and sustainability of volunteer-based peer support initiatives. Several studies have investigated volunteer motivation, highlighting the benefits of helping others, personal growth, and finding meaning in their contribution [22–25]. Conversely, reasons for disengagement with volunteer work include lack of competence, relatedness, and autonomy, as well as experiencing a high emotional cost [23, 26]. Our previous study on hematological volunteer patient peer supporters highlights several motivational factors, including a desire for personal growth, and the aspiration to inspire hope among those experiencing despair [27].

Studies investigating the motivation for peer support in the context of cancer report on motivation either before or after the peer support experience [28, 29]. To our knowledge, only one study has investigated how motivation changes over time among cancer caregivers volunteering as peer supporters [30]. Understanding how motivation evolves is crucial as it can inform strategies to sustain volunteer engagement and effectiveness. Therefore, this study aimed to explore the motivation for becoming a peer supporter and how motivation changes over time in caregivers of patients undergoing treatment for hematological malignancies.

## Methods

### Study design

This qualitative longitudinal study involved individual semi-structured interviews at two time points:

1. Just after certification as peer supporters but before starting the peer support program.
2. At the 6-week mark of their 12-week peer support program.

The interpretive phenomenological analysis (IPA) was applied for defining key findings presented as themes [31, 32].

### “The FAMCARE study”

The participants in this study were volunteer peer supporters called family caregiver ambassadors (FCA), who participated in the FAMCARE study investigating a support intervention for family caregivers [19]. The intervention consisted of 12 weeks of individualized support provided by certified FCAs to caregivers of newly diagnosed patients with hematological cancer. Following the patient’s diagnosis, their caregivers were offered peer support. If accepted, FCAs were matched with the caregiver based on criteria such as similar cancer type, gender, age, and life situation. During the 12 weeks, peer support was delivered through personal meetings, telephone calls, text messages, or video chats. All FCAs completed an online preparatory course and attended regular network meetings every 6–8 weeks for supervision and exchange of experiences with other FCAs. The preparatory course addressed the peer-to-peer role, psychological issues, and communication skills. A more detailed description is provided elsewhere [19].

### Recruitment and participants

Participants were recruited from the FAMCARE study at the Department of Hematology, Copenhagen University Hospital (May to June 2020), using purposive and convenience sampling. Caregivers of hematologic cancer survivors were eligible if they were 18 years or older, were able to read and speak Danish, and had consented to become FCAs. All 22 eligible FCAs were approached by CBM, and 11 agreed to participate, providing written informed consent before the first interview. Participants should have participated in the first interview and been active FCA providing peer support before being eligible for the second interview.

### Data collection

Eighteen semi-structured interviews were carried out by experienced clinical nurse specialists (CBM and CV) between July 2020 and January 2021. Due to the restrictions of Covid-19, the interviews were conducted by telephone. All participants were interviewed at T1 (*n* = 11), and only those who had been actively involved by providing peer support to a new caregiver during the study period could contribute and participate at T2 (*n* = 7).

During the interviews, participants were asked to describe their motivation for volunteering as a FCA to new caregivers, using the open-ended questions: “Can you describe your initial motivation to become a family caregiver ambassador?” followed by questions of “what” and “how” to further clarify their responses (Table 1). The average duration of the interviews for the first (*n* = 11) and second time points (*n* = 7) was 37 min (24–79 min.) and 26 min (17–37 min.), respectively.

**Table 1:** Interview guide

First interview			
Topic	Aim	Questions	Follow-up questions
First interview Focus on the phenomenon	To identify and describe the perceived motivation for engaging in the role of a new volunteer caregiver ambassador to new caregivers	I would like to ask you to reflect on and describe your experience of the motivation for becoming an ambassador?	Could you describe the significance of…? Could you elaborate further? Can you provide more detail/elaborate on (..)? What was your experience/feeling? You previously described(..) could you explain how it is significant?
Second interview			
Topic	Aim	Questions	Follow-up questions
Second interview Focus on the phenomenon and how it might change over time	To identify and describe the perceived motivation as a volunteer caregiver ambassador to new caregivers, and how it is influenced during an active engagement	Can you describe your experience of motivation for the role as a caregiver ambassador now that you are in an active engagement? Could you describe if and how your motivation has changed? Can you describe how you have experienced your motivation increasing or decreasing in the role?	Could you describe the significance of… Could you elaborate further? Can you provide more detail/elaborate on (..)? What was your experience/feeling? You previously described (..) could you explain how it is significant?
Focus on retention through motivation		Right now, can you describe how you feel about your motivation to engage in another process?	
Closing remarks		Is there anything we haven’t covered that you think is important regarding your motivation for the role?	

### Data analysis

The interviews were audio-recorded and transcribed verbatim. The analysis was conducted in Microsoft Word by CBM, IHN, and CV using a hands-on approach to coding, categorizing, and interpreting the data directly within a text document, following the guidelines of IPA [32]. The purpose of IPA is to explore participant experiences [31, 32], integrating phenomenological emphasis on individual descriptions, with hermeneutic interpretation [32] making it well-suited to explore FCAs motivation. The analysis entailed a collaborative four-step process. The initial two steps were conducted independently to minimize biases, by CBM, IHN, and CV, with subsequent discussions to refine understanding of the phenomenon. Researcher triangulation ensured credibility, with final validation of the thematic structure by all authors [33].

The analysis was carried out in four steps. First, each interview transcript was thoroughly reviewed to capture the essence of the data by CBM, IHN, and CV. Then, the transcripts were divided into meaningful units to facilitate analysis. The themes identified in the first step were then synthesized across all transcripts. Third, emerging themes were identified and explored through an iterative analysis process, examining connections between themes, initial notes, and textual expressions. Fourth, the identified themes were categorized and labeled based on their content, providing a structured representation of participants’ experiences by all authors. Transcripts were collaboratively translated into English by all authors to ensure accuracy, with ongoing discussions to maintain fidelity to the original wording.

## Results

Participants were male (*n* = 3) and female (*n* = 8), aged 24–73 years (mean age = 50), with educational backgrounds ranging from short-length (2–2.5 years, e.g., professional training or technical education, *n* = 2), to medium-length (3–4 years, e.g., bachelor’s degree, *n* = 4), to higher education (> 5 years, e.g., advanced degrees, *n* = 5) (see Table 2).

**Table 2:** Participant characteristic

Characteristic	Total n = 11 n (%)
Age in years**,** mean (range)	50 (24–73)
Sex, female	8 (73)
Relation to the patient	
Spouse*	7 (64)
Parent^§^	2 (18)
Sibling	1 ( 9)
Adult child^+^	1 ( 9)
Education	
Short length (2–2.5 years)	2 (18)
Medium length (3–4 years)	4 (36)
Higher education (> 5 years)	5 (46)
Years since the patient was diagnosed, mean (range)	6 (1–16)
Diagnosis of the patient	
Multiple myeloma	2 (18)
Acute leukemia	5 (46)
Chronic leukemia	2 (18)
Myelodysplastic syndrome	1 (9)
Lymphoma	1 (9)

^*^Two spouses did not participate in the second interview

^§^One mother did not participate in the second interview

^+^Did not participate in the second interview

Of the 11 participants, seven were matched with a new caregiver and actively involved in providing peer support contributing data to the second interview timepoint. The four unmatched FCAs who did not participate in the second interview included one adult child, one mother, and two spouses to former patients with multiple myeloma, acute myeloid leukemia, and chronic myelomonocytic leukemia.

**Fig. 1:**
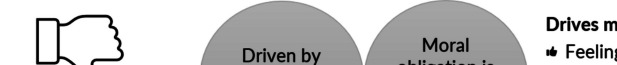
Motivation for voluntary peer support for new family caregivers

The motivation and expectations for participating in a voluntary peer-to-peer support program as an FCA center on four main themes: “Driven by past experiences”, “Moral obligation is intrinsic to identity”, “Meaningful use of personal experiences”, and “Guiding new caregivers” (see Fig. 1).

### Driven by past experiences

The motivation to become a FCA for new caregivers was described from various perspectives. Personal experiences as caregivers, including feelings of isolation, loneliness, and a lack of genuine understanding from their own families and friends, prompted a desire to provide guidance and counseling to other caregivers.

I also felt that I didn’t have much support as a caregiver while my husband was ill. So, it pushed me internally, seeing how important it was, and reminded me of how lonely one can be as a caregiver. (Id 9)

They reflected on their past experiences of balancing the desire to avoid burdening their families against their need to have someone who truly understood them to talk to. Drawing from these personal insights, they were motivated to engage in their role, striving to provide impartial support, genuine empathy, and meaningful assistance to fellow caregivers.

Some people prefer not to burden those closest to them because they already have enough on their plate. Therefore, it can be comforting to have a third-party available who neutrally offers support, without them having to worry about upsetting me. After all, I’m not upset because I’ve chosen to make myself available. (Id 2)

Some FCAs were motivated by their own experiences of receiving support from other caregivers and engaging in waiting room conversations during the treatment trajectory. Thus, having benefited from peer support themselves, they were encouraged to participate as a FCA and provide peer support in a more formalized role.

Furthermore, some FCAs having received significant support from their family and friends recognized the value of a strong social network. This sense of gratitude motivated them to become FCAs, intending to give back and share their experiences with others.

I had a good support network, but not everyone has that. It has been an eye-opener for me regarding how important family truly is and how much we can give to each other. If there’s anyone out there who doesn’t have a close family or support, I’d like to support them. (Id 3)

During their active role as FCA, this initial motivation based on their previous experiences as caregivers did not change.

### Moral obligation is intrinsic to identity

Supporting others was an ethical commitment for the FCAs, linked to their self-perception. They were motivated by a desire to help those in challenging situations whenever the opportunity arose. Volunteering and helping others were integral to their identity and way of living.

We have this strong motivation to help. It’s just deeply ingrained in me, as it always has been, to provide compassion and support to another person. (Id 7)

Some FCAs also described volunteering whenever possible as a moral duty.

I believe that if someone can offer something voluntarily, they have an obligation to do so. (Id 1)

However, the volunteering work was also expected to bring happiness and well-being to the FCAs themselves.

You receive so much love and response in return. Yes, fundamentally, it’s motivating, at least for someone like me, to feel that what I do is well received and appreciated. (Id 2)

This inherent motivation and sense of duty to volunteer remained constant throughout the peer-support experience, even if they did not receive gratitude from the caregiver. The FCAs continued to believe it was the right thing to do.

### Meaningful use of personal experiences

FCAs were motivated by the hope that their experiences would help others and, in the process, help them reconcile their own experiences and find inner peace in their new situation.

You seek purpose. It’s essential that your experiences aren’t in vain or merely stored grief, but rather lessons we can share to help others. When others find relief or understanding through our shared knowledge, suddenly, everything falls into place. (Id 11)

During the active role, the FCAs needed to feel that their efforts were aligned with the caregiver’s needs to maintain their motivation.

Sometimes, she was really frustrated when we talked, but afterward, she’d say, “Oh, it was good that we talked.” And that makes you really happy and strengthens your motivation, knowing that you’re helping others. Because if it were the opposite, where you couldn’t feel her or sense it, it would have been really difficult and demotivating. (Id 3)

The FCAs expressed a need to feel that their unique experiences were valued. If they began to doubt the meaningfulness of their role, their motivation waned. In such instances, participation in FCA network meetings proved beneficial, as one FCA described:

I couldn’t really feel I was making a difference. But fortunately, it was just incredibly rewarding to turn the situation around at the networking meeting and get the perspectives of other FCAs. It reignited my motivation. (Id. 1)

For some FCAs, using their own experiences to support the new caregiver became therapeutic and further increased their motivation.

I noticed it became somewhat therapeutic for me, like peeling off a band-aid, but instead of feeling upset, I found myself able to reflect on it in a new, positive light. (Id. 9)

### Guiding new caregivers

Helping new caregivers was a significant source of motivation for the FCAs, closely linked to the previous theme as it added value to their own experiences. They were motivated by a desire to support those overwhelmed by life’s challenges. Becoming a caregiver to a person with a hematological malignancy often involves managing numerous practical tasks, which can be overwhelming. The FCAs aimed to help new caregivers by explaining the treatment process, offering guidance on various practical tasks, and outlining available support options. This help could include nutrition advice to address the patients’ appetite loss and answering questions about examinations and treatments.

During that period, she was feeling overwhelmed by the situation, and we discussed the option of taking sick leave, which she eventually decided to do. I think that helped a lot, and I could sense that I was able to support her. (Id 6)

The FCAs were also driven by a desire to address the psychosocial concerns faced by new caregivers. Having experienced a lack of support from the healthcare system themselves, FCAs aimed to assist new caregivers in navigating the initial shock of diagnosis and managing the challenges inherent in caregiving for a person with a life-threatening illness. This involved helping them express the difficulties of being in crisis and offering guidance on coping. Their goal was to close the support gap in the healthcare system by serving as a dependable resource, assisting new caregivers navigate their emotional challenges, and validating their feelings and experiences:

To address what I perceive as a need for having a backup, for having someone to talk to, I find it important. I believe the current established treatment system may lack the time and resources to address this adequately. (Id 8)

During peer-support sessions, most FCAs found that being a “backup” person to address psychosocial concerns and sharing their experiences and understanding of the caregiver role significantly helped new caregivers, which further increased their motivation.

### Summary of results

The primary factor in maintaining motivation within the FCA role was the sense of making a difference. Their motivation decreased when they felt they were not positively impacting the new caregiver. Conversely, expressions of gratitude from caregivers, coupled with the perception that their support did not overly consume their time, either maintained or increased their motivation to proceed in the FCA role.

Participating in FCA networking meetings also helped to reinforce their motivation, particularly during times of uncertainty about the value of their assistance to caregivers. These meetings provided a platform to share experiences and discuss the challenges and benefits of the FCA role.

## Discussion

To our knowledge, this is the first qualitative longitudinal study investigating the motivation of FCAs during a peer support intervention. The findings provide new insights into how FCA’s motivation evolves from their initial engagement in peer support to their support throughout the peer support process. Prior to volunteering for peer support, FCAs were primarily motivated by a sense of moral duty intertwined with their identity, shaped by their past experiences with support during their own caregiver journeys. Additionally, FCAs were driven by a desire to utilize their personal experiences meaningfully while helping others. This initial motivation could either strengthen or diminish during peer support, influenced by the positive feedback received from new caregivers and their participation in FCA network meetings.

Snyder and Omoto [34] describe in their “Volunteer Process Model” how the specific context of volunteering shapes individual motivationKlik eller tryk her for at skrive tekst.. This model divides volunteering into three phases: *antecedents*, *experience*, and *consequences*, suggesting that the duration of volunteering depends not only on individual motivation but also on interpersonal experiences and interactions with the organization throughout the volunteering period. Our findings resonate with this perspective, as the FCAs emphasize not only the influence of their past individual support experiences on their motivation but also the significance of their interpersonal dynamics with the new caregiver. For instance, their perception of making a meaningful difference for the new caregiver during the support process affects their motivation, as does their participation in ambassador network meetings facilitated by the “FAMCARE” study.

In another study of volunteers in a humanitarian non-profit organization in the UK [35], interpersonal interactions were observed to play a crucial role in motivating volunteers. Similar to our findings, volunteer caregivers’ ability to see how their work helped others encouraged them to remain in their roles longer or devote more time [35]. This emphasizes the importance of feedback from the caregiver receiving support, which also plays an important role in the recovery trajectory of the caregiver support providers. The social psychologist Frank Riessman describes “The Helper Therapy Principle” [36], suggesting that individuals who help others can find personal fulfillment and growth. Similarly, our study found that engaging in the role of an FCA could have a therapeutic effect, implying a symbiotic relationship in which helping others also strengthened one’s well-being.

Previous research has identified various reasons why people volunteer, often categorizing motivational factors as intrinsic or extrinsic [37]. Likewise, our results show that FCAs were primarily driven by intrinsic motivations, such as the desire to help others and the personal satisfaction derived from feelings of purpose, meaningfulness, and altruism. Our findings suggest that extrinsic motivation, such as financial rewards or social recognition, does not significantly influence the FCAs’ motivation as peer supporters. One possible explanation is that FCAs, being caregivers of former patients themselves, are particularly motivated by altruism and empathetic emotions focused on the well-being of others [37].

Skirbekk et al. [38] found that a key motivation for patients and caregiver peer supporters was the meaningful use of their own experiences. This aligns with our findings of both the initial motivation for becoming a FCA and throughout their support role. We found that the wish to help fellow family caregivers was a significant source of motivation for FCAs, closely linked to their intention to use their own experiences. However, if FCAs felt unable to utilize their experiences to provide support, their motivation was negatively affected, leading to doubts about their role. Similarly, a study by Vodermaier et al. [39] identified both benefits, such as a greater sense of meaning and purpose from helping others, and stressors, including concerns about role performance, among prostate cancer and caregiver peer navigators. This underscores the importance of offering role competencies, supervision, and a supportive social network for peer supporters both initially and throughout their engagement to maintain intrinsic motivation and competencies.

In a previous study, we investigated the motivation of patient peer supporters treated for acute leukemia [29]. We found that former patients were motivated by using their own experiences to help new patients and simultaneously move forward with their own life by transition from being identified as a patient to discovering a new survival identity beyond the disease. In the current study among caregivers as peer supporters, we found the same motivation for using their own experiences, but we did not find the desire to transcend away from the caregiver identity as a motivational factor. This may indicate that caregivers have different concerns and struggles compared to patients. It is important to consider the various roles and experiences of patients and caregivers to understand nuances and drivers related to the motivation for becoming a peer supporter, as well as the benefits derived from providing peer support in both groups.

### Strength and limitations

This study is the first to investigate motivation among volunteer caregiver peer supporters in hematology, providing novel insights into how motivation evolves over time. The validity of the analysis is strengthened by IPA [32], ensuring robustness by maintaining coherence between interview questions, participants’ descriptions, and findings. Researcher triangulation further reinforced the credibility of the findings. However, there are limitations. Participants were recruited from an ongoing feasibility study (FAMCARE), using purposive and convenience sampling. While purposive sampling allowed us to select participants with relevant experience in peer support, the results may not be fully representative of the broader population of peer supporters which may limit the generalizability of the findings. However, to capture participants’ initial, unaffected motivation, the first interviews were conducted prior to their first formal peer support experience. This timing ensured that their motivation was not yet influenced by any positive or negative experiences associated with the role. In contrast, the second interview, conducted after they had gained experience as ambassadors, provided deeper insight into the factors influencing their motivation to remain in the peer support role. Additionally, only half of the 22 eligible participants consented to this qualitative study, and we have no data on non-participants, who might differ from those who did participate. Only seven participants were matched with new caregivers and actively involved in providing peer support, which affects the depth and breadth of the longitudinal investigation. This limitation highlights the need for further research to explore the long-term experiences of motivations of a larger group of volunteer peer supporters.

## Conclusion

A sense of moral obligation initially drives the former caregiver towards the FCA role. However, to maintain motivation for the voluntary role over time, they find it crucial to use their own caregiver experiences in a meaningful way. A key finding emphasizes the importance of organizing networking meetings for FCAs. These meetings facilitate the exchange of experiences, enabling participants to discuss both the challenges and rewards of their roles with fellow FCAs. Such interactions serve as a significant motivator for sustaining peer support efforts and provide opportunities to reignite motivation if it diminishes.

## Data Availability

The data that support the findings of this study are available from the corresponding author upon reasonable request.
